# An Update on Novel Drug Delivery Systems for the Management of Glaucoma

**DOI:** 10.3390/pharmaceutics17081087

**Published:** 2025-08-21

**Authors:** Harshilkumar S. Jani, Ketan Ranch, Radhika Pandya, Yashkumar Patel, Sai H. S. Boddu, Amit K. Tiwari, Shery Jacob, Haya Khader Ahmad Yasin

**Affiliations:** 1 Department of Pharmaceutics, L. M. College of Pharmacy, Navrangpura, Ahmedabad 380009, Gujarat, India; harshil.jani@lmcp.ac.in (H.S.J.); radhika.pandya@lmcp.ac.in (R.P.); yash.patel@lmcp.ac.in (Y.P.); 2 Research Scholar, PhD—Pharmacy, Gujarat Technological University, Ahmedabad 382424, Gujarat, India; 3 Department of Pharmaceutical Sciences, College of Pharmacy and Health Sciences, Ajman University, Ajman P.O. Box 346, United Arab Emirates; s.boddu@ajman.ac.ae; 4 Center of Medical and Bio-Allied Health Sciences Research, Ajman University, Ajman P.O. Box 346, United Arab Emirates; 5 Department of Pharmaceutical Sciences, College of Pharmacy, University of Arkansas for Medical Sciences (UAMS), Little Rock, AR 72205, USA; atiwari@uams.edu; 6 Department of Pharmaceutical Sciences, College of Pharmacy, Gulf Medical University, Ajman P.O. Box 4184, United Arab Emirates; dr.sheryjacob@gmu.ac.ae

**Keywords:** nanocarriers, ocular drug delivery, glaucoma, hydrogel, contact lens

## Abstract

Glaucoma is recognized as a chronic optic neuropathy marked by progressive optic nerve degeneration, loss of retinal ganglion cells (RGCs, the neurons responsible for transmitting visual information from the eye to the brain), disruptions in optic disc blood supply, and changes in glial cell activation. It ranks as the second most prevalent cause of irreversible visual impairment worldwide and is a resultant of increased intraocular pressure (IOP). Addressing this condition proves complex due to the inherent hindrances posed by ocular barriers, which curtail the entry of drugs into the eye. Diverse carriers such as inorganic nanoparticles, polymeric nanocarriers, hydrogels, and contact lens-based systems with distinct physical and chemical attributes are being studied for drug delivery. They have shown enhanced ocular drug bioavailability through higher penetration across ocular tissues, prolonged retention in the precorneal space, sustained drug release, and targeted delivery to specific tissues. These ingenious delivery systems can be deployed through various administration routes—intravitreal or periocular injections or systemic administration—enabling the drugs to reach affected areas, aiding in the regeneration of compromised optical nerves. This review presents a comprehensive exploration of contemporary strides in ocular delivery formulations pertaining to glaucoma. This encompasses an examination of various nanocarrier typologies, delivery routes, in vitro and in vivo effectiveness, clinical applicability, and a forward-looking perspective into potential future developments.

## 1. Glaucoma and Challenges Associated with Its Management 

### 1.1. Management of Glaucoma

**Figure 1:**
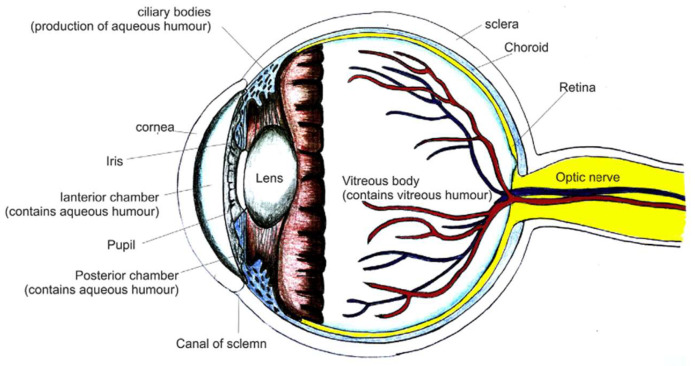
Healthy human eye.

**Figure 2:**
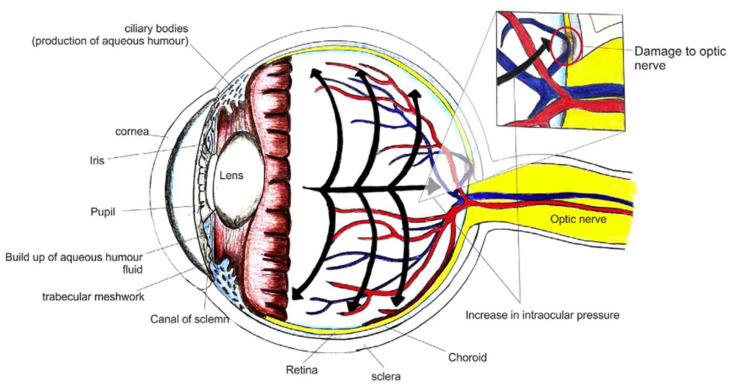
Glaucomic human eye.

Glaucoma stands as a persistent eye condition characterized by the dysfunction of the optic nerve, leading to a gradual loss of the visual field. This results from the progressive demise of retinal ganglion cells (RGCs), making it the second leading cause of blindness globally, following age-related macular degeneration. According to the 2020 ‘World Report on Vision’ by WHO, an estimated 76 million individuals are currently affected by glaucoma, with a projected alarming increase of 1.3 times by the year 2030 [1]. The understanding of glaucoma’s mechanisms and associated changes lays the groundwork for effective therapeutic strategies. Glaucomatous eyes experience pressure build-up, causing alterations in the optic nerve. The fluid flow across the anterior and posterior chambers and the impact of elevated intraocular pressure (IOP) and oxidative stress on Muller cells and astrocyte activation lead to damage of the optic nerve and retinal ganglion cells [2]. This impairment disrupts the essential elements needed to preserve RGC homeostasis. Moreover, elevated IOP compromises the microvascular supply to the optic disc, potentially causing RGC ischemia [3]. Figure 1 depicts a normal healthy eye, while Figure 2 depicts the impact of increased IOP on optic nerve damage in glaucomatous eyes. 

IOP reduction serves as a pivotal measure to slow down glaucoma progression. Hence, the primary focus of treatment is on lowering IOP. The standard approach involves utilizing topical anti-glaucoma eye drops, followed by potential escalation to oral medications, laser treatments, and surgical interventions. Topical anti-glaucoma eye drops play a key role in IOP reduction, employing various mechanisms. Cholinergic agonists contract the ciliary muscle, altering lens shape for increased focusing power, while also tightening trabecular meshwork cells to facilitate aqueous humor outflow. Alpha-adrenergic agonists decrease aqueous humor production, carbonic anhydrase inhibitors lower secretion through enzyme activity reduction, and beta-adrenergic receptor antagonists diminish production with possible side effects [4,5]. Prostaglandin analogues elevate uveoscleral outflow but can trigger adverse effects. However, conventional eye drop treatments are often hindered by poor tolerance and compliance due to the need for frequent reapplication. Fixed drug combinations have been designed to enhance patient compliance and reduce preservative exposure. Injectable sustained-release formulations present another avenue to improve compliance, delivering medication over extended periods. Table 1 summarizes various treatment options for glaucoma [6].

**Figure 3:**
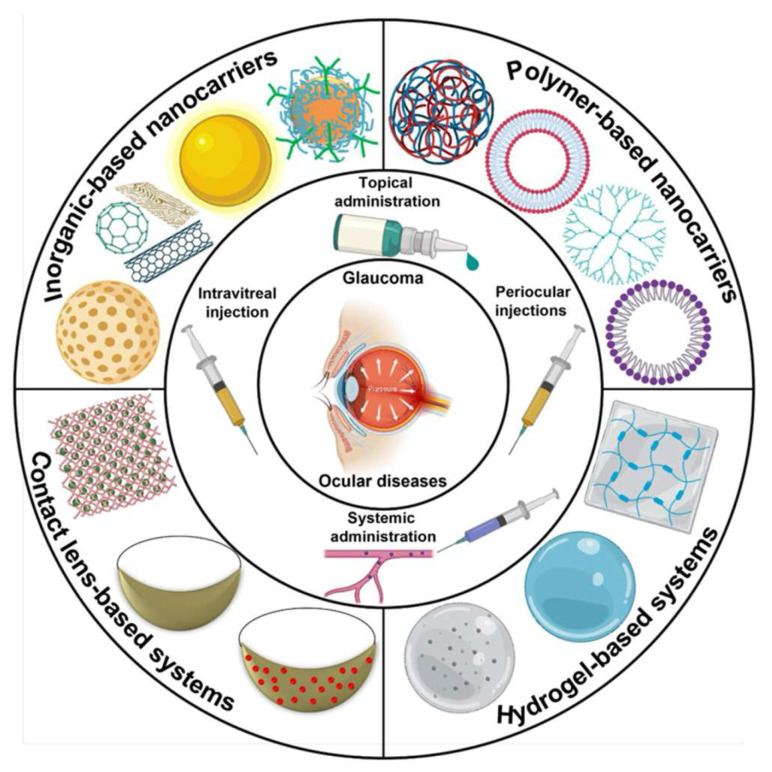
Schematic illustration of different types of nanomaterial formulations and their various ocular delivery administrations in glaucoma applications discussed in this review.

This comprehensive review focuses on the challenges associated with glaucoma treatment and the utility of various nanocarrier formulations in treating glaucoma. The discussion highlights aspects such as drug-loading capacity, both physical and biological limitations, strategies for drug delivery, and the penetration and distribution of nanocarriers in the eye. The review also underscores the major challenges, opportunities, and future prospects in the realm of nanocarrier development and their efficacious application in glaucoma treatment. This overarching goal of this review is visually summarized in Figure 3, where the outer concentric circle illustrates diverse biomaterials employed for drug formulations, the middle circle depicts administrative approaches for drug delivery, and the innermost circle represents the targeted disease. 

### 1.2. Challenges Associated with Ocular Drug Delivery

The delivery of drugs to the eye is intrinsically challenging because of several physiological and anatomical barriers that limit drug bioavailability and efficacy [17]. The cornea acts as a major barrier to drug absorption because of its tightly packed epithelial cells and hydrophobic nature, hindering the permeation of hydrophilic drugs [18]. Similarly, the conjunctiva, a highly vascularized membrane lining the inner surface of the eyelids and covering the sclera, facilitates drug absorption into the systemic circulation, thus reducing the drug amount available to reach the target tissues within the eye [19]. The blood–aqueous barrier (BAB), formed by tight junctions between the non-pigmented ciliary epithelium cells, restricts the entry of many substances from the bloodstream into the aqueous humor, further limiting drug penetration [20]. Furthermore, the lacrimal system, responsible for tear production and drainage, efficiently removes topically applied drugs from the ocular surface, reducing the contact time and overall drug absorption [21]. Novel drug delivery systems, such as nanoparticles, liposomes, and novel ocular inserts, are being explored to overcome these barriers and enhance drug penetration into the eye [22]. Despite the potential benefits of these advanced drug delivery systems, challenges remain in terms of toxicity, stability, scale-up, and clinical performance [23]. Systemic drug administration to reach the anterior segment of the eye is hampered by the BAB, which is formed by inner ciliary epithelia, endothelia around the iris, and ciliary muscle capillaries, thereby greatly diminishing ocular bioavailability of many drugs [24,25]. Modification of drugs to improve their permeability, developing formulations that prolong contact time with the ocular surface, and employing targeted drug delivery systems are crucial strategies for improving the effectiveness of glaucoma medications [26].

### 1.3. Challenges and Advances in Local Drug Delivery for Glaucoma

The pharmacological management of glaucoma often involves the long-term use of topical eye drops, which can lead to a range of local and systemic side effects, and the financial burden of medications can also be a significant barrier to treatment adherence [27]. Topical glaucoma medications, such as prostaglandin analogs, beta-blockers, and alpha-adrenergic agonists, are associated with various adverse effects, including ocular irritation, stinging, redness, blurred vision, and allergic reactions [28]. Prostaglandin analogs, while highly effective in lowering intraocular pressure, can cause changes in iris pigmentation, eyelid skin darkening, and periocular tissue atrophy, which can be cosmetically concerning for some patients. Clinical studies have revealed that the use of topical prostaglandin analogues only results in a roughly 50% reduction in visual field progression when compared to a placebo. A controlled randomized trial involving glaucoma patients treated with latanoprost 0.05% eye drops or a placebo demonstrated that after 24 months, the mean IOP reduction was 3.8 mm Hg in the latanoprost group compared to 0.9 mm Hg in the placebo group [29]. Although current glaucoma treatments exhibit the capability to effectively lower IOP, they often struggle to consistently regulate the IOP diurnal curve. Studies have indicated that an increase in IOP fluctuations corresponds to an elevated risk of visual field deterioration. Additionally, prolonged IOP fluctuations exceeding 24 h have been implicated in glaucoma progression. Therefore, continuous monitoring of IOP throughout a 24-h timeframe could offer a more accurate assessment of factors influencing IOP variability [30,31]. Beta-blockers, once a mainstay of glaucoma therapy, can cause systemic side effects such as bradycardia, hypotension, and bronchospasm, particularly in susceptible individuals with underlying cardiovascular or respiratory conditions [28]. Alpha-adrenergic agonists can lead to systemic effects such as dry mouth, fatigue, and dizziness, limiting their use in certain patient populations. Carbonic anhydrase inhibitors can also result in side effects that some patients cannot tolerate. A substantial portion of glaucoma patients (74%) are unwilling to use eye drops, leading to exploration of alternative treatment strategies such as subconjunctival injections administered every three months [32]. Subconjunctival injections or implants have gained some traction as a less invasive and well-received option for managing and treating glaucoma [33,34]. 

The cumulative effect of these side effects can significantly impact a patient’s quality of life and willingness to adhere to the prescribed treatment regimen [28]. In many developing countries, the cost of glaucoma medications is a major obstacle to treatment adherence, as patients may need to take these medications for the rest of their lives [35]. The expense of medications can be especially burdensome for patients with limited financial resources, leading to missed doses or discontinuation of treatment, which can have devastating consequences for their vision. Finding cost-effective strategies to manage glaucoma, such as generic medications or patient assistance programs, is critical to improving access to treatment and preventing vision loss in this vulnerable population. The ideal treatment choice is also affected by the efficacy, compliance, and potential side effects of the prescribed medication [36]. For a drug to be effective, it has to lower the IOP by at least 20% [37].

### 1.4. Adherence to Treatment

Adherence to prescribed treatment regimens is a critical determinant of success in managing chronic conditions such as glaucoma, but it remains a significant challenge in clinical practice [38,39]. Glaucoma often requires long-term, if not lifelong, treatment with topical eye drops, which can be difficult for patients to adhere to consistently over time. Several factors contribute to poor adherence, including forgetfulness, complex dosing schedules, lack of understanding of the disease and treatment, side effects, and cost [27]. As glaucoma is frequently asymptomatic until significant vision loss has occurred, patients may not perceive the immediate benefits of treatment and may be less motivated to adhere to the prescribed regimen [40]. Simplifying treatment regimens, providing thorough patient education, addressing side effects, and promoting open communication between patients and providers are essential strategies for improving adherence and maximizing treatment outcomes in glaucoma [41]. Studies have shown that interventions such as medication reminder systems, adherence aids, and motivational interviewing can be effective in improving adherence to glaucoma medications [42]. Qualitative studies reveal that a patient’s knowledge of their disease, personal biographies, and living conditions affect adherence to glaucoma medication [43]. Furthermore, creating a supportive and collaborative environment where patients feel empowered to actively participate in their care can significantly enhance adherence and improve long-term outcomes [44]. Understanding adherence barriers is necessary to determine strategies to improve medication adherence and achieve optimum outcomes [45]. 

## 2. Novel Drug Delivery Systems for Glaucoma Management 

In the context of ocular drug delivery, approximately 90% of current formulations are in the form of solutions, primarily eye drops. However, ocular delivery presents significant challenges due to the rapid elimination rate resulting from tear turnover and drainage through the nasolacrimal duct. Moreover, ocular pharmaceutics face additional hurdles that can undermine patient compliance and treatment efficacy. An ideal ocular formulation should exhibit sustained drug release, enhanced penetrability and tissue residence time, isotonicity, biodegradability, biocompatibility, and minimal irritation. In recent years, there has been a remarkable surge in the development of advanced ocular drug delivery systems that offer controlled and sustained release of medications for glaucoma treatment. Nanocarriers can effectively address the challenges of low drug bioavailability and subsequent fluctuations in IOP. Through their controlled delivery mechanism, these carriers ensure a consistent and extended release of the medication, promoting better therapeutic outcomes over prolonged periods. The delivery systems include inorganic nanoparticles, polymer nanoparticles, vesicular systems, and miscellaneous systems such as hydrogels and contact lenses. Ensuring compatibility between drug and carrier chemistries is crucial in nanocarrier-based drug delivery. This guarantees stable drug loading and carrier-drug linkages that are not only stable but also responsive to the local tissue environment and pathological changes, such as pH fluctuations during ocular inflammation [46,47,48]. Notably, inorganic nanoparticles such as iron oxide, silver, gold, carbon-based nanomaterials, and mesoporous silica-based nanoparticles have been utilized for ocular drug delivery due to their unique attributes. Being inorganic nanoparticles, these systems provide more precise size and shape control, a large volume-to-surface area ratio, versatile surface functionality, biocompatibility, enhanced dissolution rates, penetration capabilities, efficient distribution, external stimuli-based accumulation, and the capacity to adhere to cell membranes [49]. Similarly, polymer-based nanocarriers, like nanocapsules, nanoparticles, micelles, liposomes, and dendrimers, have gained significant attention. These carriers have been extensively investigated for their ability to transport diverse therapeutic agents, ranging from drugs and growth factors to proteins, genes, and peptides. Other biomaterials such as hydrogel-based formulations, contact lenses, and implants have also undergone substantial research as promising approaches for controlled and direct drug delivery (Table 2). 

Ideally, sustained-release drug delivery systems operating over extended periods (weeks, months, or even years) can maintain an optimal drug concentration at the target site. This approach enhances patient adherence to therapy, reduces systemic side effects, and provides better IOP control over a 24-h duration. Integrating sustained-release technologies into glaucoma treatment would offer substantial advantages to both patients and healthcare professionals. However, beyond usefulness and safety considerations, cost-effectiveness must also be addressed when introducing such products into the market [50]. For instance, the Ocusert^®^ ocular insert was introduced commercially in 1975, designed to provide controlled, zero-order release of pilocarpine over a week. Despite its initial success, it was withdrawn from the market in 1993 due to inadequate IOP reduction and excessive side effects in comparison to traditional pilocarpine eye drops. The Ocusert^®^ had other drawbacks, including difficult insertion, expulsion from the eye, and drug leakage. When new technologies are brought to market, their efficacy and cost are generally evaluated against existing commercial products. To be both medically effective and commercially viable, sustained-release products must surpass available options in terms of safety, convenience for patients and clinicians, and competitive pricing [51]. Achieving precise and sustained delivery of therapeutic biomolecules to specific tissues or cells serves several critical purposes, such as prolonging therapeutic action in the desired area, promoting uniform distribution of therapeutics, minimizing nonspecific binding to surrounding tissues, and ultimately reducing the required therapeutic dose [51].

### 2.1. Inorganic Nanoparticles for Glaucoma Management

Inorganic nanoparticles offer several advantages, such as controlled structural dimensions, precise size, diverse shapes, specific surface area, and versatile surface functionality. These characteristics infuse them with the capability to surmount challenges like limited solubility, efficient penetration, uniform distribution, accelerated dissolution kinetics, and adherence to cell surface membranes. Ensuring a consistent nanoparticle size and maintaining suspension in a suitable liquid medium are critical factors for optimizing drug delivery. Typically, surface modifications, often involving chemical alterations, are achieved through the application of surfactants, resulting in nano-suspensions. Moreover, recent advancements allow for nanoparticle surface modification during the preparation phase, eradicating the need for toxic substances. The dispersal media generally encompass water or organic solvents, such as polyethylene glycol and various oils. Among the diverse array of nanoparticles, including iron oxide, silver, gold, silica, and carbon-based nanomaterials, some serve as promising vehicles for therapeutic delivery [52,53,54].

#### 2.1.1. Gold Nanoparticles

Gold nanoparticles (AuNPs) have gained significant attention in ophthalmology due to their ability to act as both diagnostic and therapeutic agents. Their high surface area-to-volume ratio allows for efficient drug loading and controlled release. AuNPs can be functionalized with various molecules, such as drugs, targeting ligands, and polymers, to enhance their therapeutic efficacy and specificity [55,56]. AuNPs can be engineered to target specific tissues within the eye, such as the trabecular meshwork, which is crucial for managing intraocular pressure in glaucoma patients. Sonntag et al. evaluated the optimal size for targeting the trabecular meshwork by using AuNPs of various sizes (5, 60, 80, and 120 nm) with either a bare surface (AuNPs) or coated with hyaluronic acid (HA-AuNPs). This comparison was made regarding their colloidal stability, distribution in the anterior chamber of the eye ex vivo, and cellular uptake in vitro. Studies have shown that HA-AuNPs exhibit exceptional colloidal stability and preferential accumulation in the trabecular meshwork, minimizing off-target effects in other ocular tissues. The study found that gold NPs, particularly those with a diameter of 120 nm and coated with HA, exhibited exceptional colloidal stability and the highest accumulation in the trabecular meshwork. This characteristic enhances their potential for delivering encapsulated anti-glaucoma drugs effectively [57]. Contact lenses embedded with AuNPs have been explored as a novel drug delivery platform. These lenses can release drugs such as timolol over an extended period, improving bioavailability and reducing the need for frequent administration. Studies have shown that AuNP-infused contact lenses enhance drug accumulation in the ciliary muscles, leading to sustained IOP reduction. AuNP-laden contact lenses have gained attention in the past few years due to the increase in drug loading and sustained drug delivery. Timolol [58], bimatoprost [59], and travoprost [60] have been delivered using AuNP-laden contact lenses.

AuNPs have also been investigated for topical delivery to the posterior segment of the eye, which is challenging due to the ocular immune privilege and anatomical barriers. Hyaluronic acid-coated AuNPs have shown promise in bypassing these barriers, achieving higher distribution in the retina and retinal pigment epithelium compared to uncoated AuNPs. This advancement opens new avenues for treating glaucoma and other retinal diseases [61]. Notably, the distinctive optical properties of AuNPs, which are influenced by their size and shape, coupled with their low cytotoxicity, remarkable stability, and facile surface modification, position them as highly appealing nanocarriers in the realm of therapeutic delivery and nanomedicine. Moreover, surface functional groups, charge, polarity, and ligand interactions play pivotal roles in dictating the physicochemical attributes of AuNPs, which in turn significantly contribute to their efficacy in targeted drug delivery. For instance, AUNPs can be functionalized with glaucoma drugs to enhance delivery to specific ocular tissues like the trabecular meshwork. The controlled release of drugs can be achieved through covalent binding or supramolecular interactions between drug molecules and the surface of AuNPs, enabling the controlled delivery of therapeutic agents within the ocular system. AuNPs-based drug delivery systems offer the prospect of precise targeted delivery and controlled release of drugs, accompanied by sensitive bioimaging modalities that facilitate early disease diagnosis [62,63]. AUNPs showed antioxidant and anti-inflammatory properties, which may help in protecting RGCs, the cells that die in glaucoma [55]. 

Kim et al. have explored the ability of AuNPs to traverse the blood–retinal barrier (BRB) and distribute within the retinal layer following intravitreal and intravenous administration. No adverse effects were observed in retinal, astrocyte, endothelial, or retinoblastoma cells [63]. Larger AuNPs (100 nm) exhibited limited crossing of the BRB, while smaller AuNPs (20 nm) successfully passed through and spread within the retinal area upon intravenous administration in C57BL/6 mice. This observation suggests the potential candidacy of smaller AuNPs for therapeutic delivery to the retinal region. Moreover, AuNPs were found to inhibit retinal neovascularization through the suppression of VEGFR-2 activation in a retinopathy of prematurity (ROP) animal model, which not only hindered angiogenesis but also prevented the auto-phosphorylation of VEGFR-2, thus mitigating the initiation of the ERK 1/2 pathway [64]. Cho et al. employed topically administered AuNPs to effectively inhibit corneal neovascularization in mice, substantially reducing neovascularized areas by 39% and decreasing VEGFR-2 expression, consequently curbing inflammation [65]. The utilization of inorganic nanoparticles presents a promising avenue for gene delivery in the treatment of eye-related diseases. AuNPs have shown remarkable potential as gene delivery carriers, facilitating significant transfection of genes into mammalian cells and subsequent gene expression. 

#### 2.1.2. Silver Nanoparticles

Silver nanoparticles (AgNPs) have a longstanding history of application in biomedicine, attributed to their antibacterial, anti-inflammatory, and antioxidant properties, as well as their potential for drug delivery. Remarkably, AgNPs possess antiangiogenic attributes, effectively curtailing the survival of retinal endothelial cells. Sondi et al. elucidated that AgNPs exert their antibacterial effect through structural alterations and membrane damage, ultimately leading to apoptosis of bacterial cells [66]. McQuillan et al. postulated that AgNPs engage with both the outer and inner bacterial cell walls, prompting the entrance of silver ions into the cell and orchestrating transcriptional regulation [67]. Additionally, the interaction between positively charged ions and negatively charged bacterial surfaces results in the disruption of electron transfer and impedes the upregulation of ATPase translocation [68]. Numerous studies have validated the safety and biocompatibility of AgNPs, though caution is warranted regarding large particle sizes and extreme concentrations that could potentially induce cytotoxicity and genotoxicity. Intriguingly, the topical administration of AgNPs in the ocular regions of mice and rabbits has been found to be compatible and well tolerated, underscoring their potential for ophthalmic applications. Notably, AgNPs and silver nitrate have found utility in ophthalmology as agents to prevent ophthalmia neonatorum [69,70].

**Figure 4:**
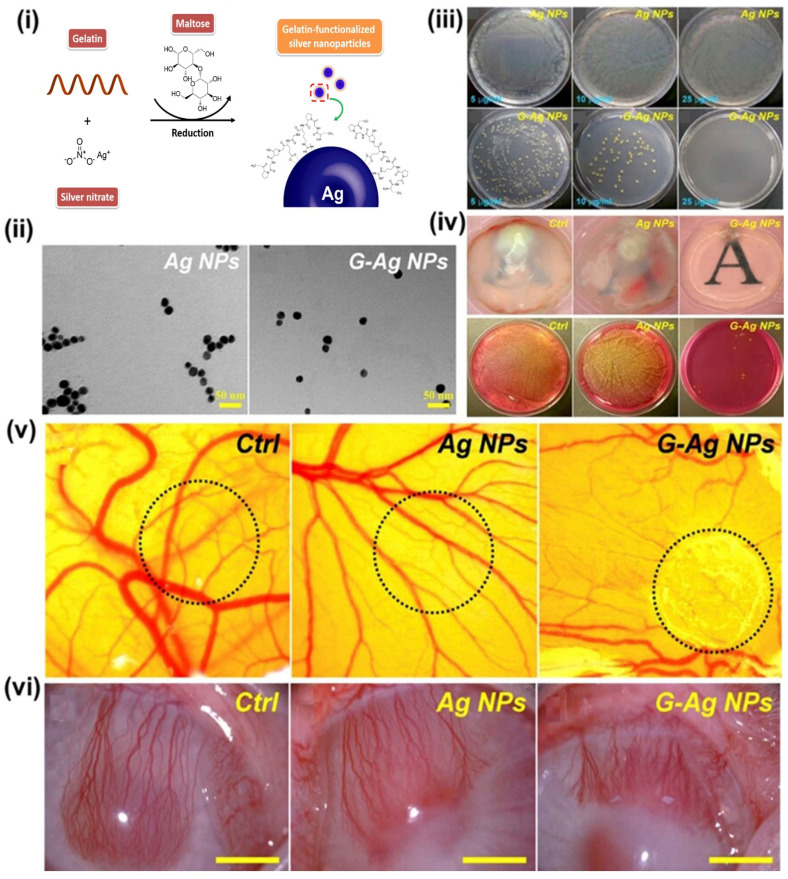
Inorganic nanoparticle nanomaterials in ocular applications: Silver nanoparticles (AgNPs) and gelatin-modified G-Ag NPs for BK treatment; (**i**) schematic diagram of the environmentally friendly synthesis of G-AgNPs; (**ii**) TEM images of AgNPs and G-AgNPs; (**iii**) optical images of bacteria exposed with Ag NPs or G-Ag NPs with varying concentrations of 5, 10, and 25 Mg/mL; (**iv**) optical images of corneal (typescript beneath) samples of rabbit eyes at day 3 after surgery and through an experiment caused Bk and then injection of AgPs or G-Ag NPs via intrastromally; (**v**) optical images of CAM vasculature after exposure with AgNPs or G-Ag NPs at day 1; and (**vi**) Representative optical images of slit lamp of VEGF-A165-induced rabbit CNV treated with AgNPs or G-Ag NPs; Ctrl samples were treated with PBS injections only, and these images were taken at day 3 (scale bars: 2 mm). Reproduced with permission from Elsevier from Ref. [71].

Capitalizing on these advantageous properties, Luo et al. engineered gelatin-functionalized silver nanoparticles (G-AgNPs) as nanocarriers for antibacterial and antiangiogenic interventions in bacterial keratitis (BK) [71]. The synthesis of AgNPs involved a chemical reduction process utilizing silver nitrate in the presence of maltose, followed by the formulation with gelatin functionalization (Figure 4i). The original AgNPs displayed aggregation, while modification with gelatin led to particle dispersion (Figure 4ii). These G-AgNPs exhibited antibacterial efficacy against S. aureus as demonstrated by treatment on Luria broth (LB) agar plates for a day (Figure 4iii). Evaluation after rabbit corneal surgery revealed a noteworthy reduction in corneal transparency due to severe bacterial infection in the control group rabbit (Figure 4iv). Intriguingly, the intrastromal injection of AgNPs resulted in compromised tissue clarity, indicating that unmodified nanoparticles do not effectively protect against the *S. aureus* infection-induced injuries, whereas G-AgNPs injection mitigated cloudiness, resulting in improved transparency. The effect was also confirmed using corneal tissues cultured and infected with S. aureus on mannitol salt agar (MSA) plates for 24 h, showing the reduction in S. aureus colonies, which indicates the superior antibacterial activity of the G-Ag NPs. Furthermore, the angiogenic activity of both AgNPs and G-AgNPs was examined through the CAM assay, indicating that gelatin functionalization curtailed vascular network formation (Figure 4v). The in vivo antiangiogenic potential of AgNPs and G-AgNPs was corroborated in a rabbit model of experimental CNTV, affirming the antiangiogenic capabilities of Ag-NPs by inhibiting blood vessel formation (Figure 4vi). Notably, angiogenesis plays a pivotal role in the intricate process of capillary network formation within endothelial cells. In cases of diabetic patients, pathological neovascularization underpins vision loss, notably in retinopathy of prematurity (ROP) and age-related macular neovascularization [71]. Concerns regarding the toxicity of nanoparticles, particularly AgNPs, have been a significant barrier to their clinical application. Long-term exposure to AgNPs may lead to accumulation in ocular tissues, raising questions about potential adverse effects and the safety profile of these formulations [72,73]. Studies indicate that the residence time, transport, and biotransformation of nanoparticles in the body are closely related to their toxicity, suggesting that more research is needed to elucidate the implications of chronic exposure to AgNPs in humans [74]. 

#### 2.1.3. Iron Oxide Nanoparticles

Iron nanoparticles have shown promise in the field of drug delivery for glaucoma, particularly in enhancing the delivery and tracking of therapeutic agents. The use of superparamagnetic iron oxide (SPIO) nanoparticles, integrated into a polylactic acid-glycolic acid (PLGA) polymer matrix, has been explored to improve the delivery accuracy and in vivo tracking of stem cell-based therapies for glaucoma. This approach aims to remodel the trabecular meshwork (TM) and restore intraocular pressure (IOP) homeostasis, which is crucial in managing glaucoma. The integration of SPIO nanoparticles allows for magnetic manipulation, enhancing the precision of cell delivery and providing a dual-model tracking system for long-term monitoring. SPIO nanoparticles are used to label induced pluripotent stem cell (iPSC)-derived TM cells, improving delivery accuracy and tracking efficiency in vivo. The magnetic properties of SPIO allow for temporary enhancement of cell-based therapy effectiveness, aiding in alleviating glaucoma pathologies. In vitro studies indicate that SPIO nanoparticle labeling does not adversely affect cell viability or fate, ensuring the safety of this delivery method. The use of SPIO nanoparticles in stem cell-based therapies offers a promising approach for clinical translation, potentially improving the management of glaucoma by enhancing both delivery and tracking of therapeutic agents [75]. 

#### 2.1.4. Carbon-Based Nanomaterials

Carbon-based nanomaterials, particularly single-walled carbon nanotubes (SWCNTs), are gaining interest as potential platforms for drug delivery in glaucoma treatment due to their high surface area, tunable functionalization, and ability to traverse ocular barriers for sustained release and enhanced bioavailability. The use of these nanomaterials addresses the challenges posed by the eye’s complex barriers, which often limit the bioavailability of traditional antiglaucoma drugs. This innovative approach not only improves drug delivery but also enhances patient compliance by reducing the frequency of drug administration. Below are key aspects of carbon-based nanomaterials in glaucoma treatment. Carbon-based nanomaterials can penetrate the eye’s biological barriers, such as the blood–retinal and blood–aqueous barriers, which are typically challenging for conventional drugs to bypass. While there are limited in vivo glaucoma-specific studies, a 2025 review highlighted the potential of SWCNTs in ophthalmology, noting their compatibility and suitability for functionalization with therapeutic agents for improved solubility, targeting, and reduced cytotoxicity. Another review of nanoscale drug delivery systems in glaucoma therapy affirms SWCNTs as a central component alongside other nanomaterials, underscoring their relevance to anti-glaucoma drug transport across ocular barriers [76]. These nanomaterials facilitate a controlled release of drugs, maintaining therapeutic levels over extended periods and reducing the need for frequent dosing. The ability to target specific tissues, such as damaged nerves in the eye, enhances the efficacy of the treatment and minimizes systemic side effects. Despite their potential, the bioavailability of drugs delivered via carbon-based nanomaterials can still be a concern, necessitating further research to optimize formulations. While carbon-based nanomaterials offer significant advantages for drug delivery in glaucoma treatment, there are still challenges to address, such as optimizing bioavailability and ensuring safety [77,78].

#### 2.1.5. Mesoporous Silica-Based Nanoparticles (MSNs)

MSNs possess a large surface area and pore volume, which facilitate high drug loading capacity and efficient encapsulation of therapeutic agents [79,80]. The pore size of MSNs can be adjusted to accommodate different drug molecules, allowing for tailored drug release profiles [81]. MSNs are biocompatible, reducing the risk of adverse reactions when used in ocular applications. MSNs can be functionalized with various chemical groups to enhance their interaction with specific cells or tissues, improving targeting efficiency and reducing off-target effects [82]. Lai et al. fabricates pH-triggered drug-eluting contact lenses (DCLs) that incorporate large-pore mesoporous silica nanoparticles (LPMSNs) to enhance drug delivery for glaucoma treatment. The DCLs are designed to prolong the residence time of drugs in an artificial lacrimal fluid (ALF) environment at a physiological pH of 7.4. The study found that pH-triggered drug-eluting contact lenses (DCLs) combined with large-pore mesoporous silica nanoparticles (LPMSNs) significantly prolong the residence time of glaucoma drugs in an artificial lacrimal fluid environment at pH 7.4, enhancing drug delivery efficiency without the need for drug preloading. LPMSN-laden DCLs demonstrated improved drug loading capabilities at pH 6.5 due to specific adsorption, and their sustained and extended release of glaucoma drugs was successfully monitored, with no observed cytotoxicity, indicating their potential as safe and effective nanocarriers for ophthalmic drug delivery [83]. While mesoporous silica nanoparticles offer significant advantages for glaucoma drug delivery, challenges remain in optimizing their design for specific therapeutic needs. Further research is needed to explore the full potential of MSNs in clinical settings, including more extensive in vivo studies to assess their long-term safety and efficacy.

### 2.2. Polymeric Nanocarriers for Glaucoma Management

There has been an extraordinary increase in the research, clinical, and pharmaceutical fields focused on polymer-based nanocarriers for drug delivery uses. These nanocarriers utilize the unique properties of polymers, whether natural or synthetic, owing to their biocompatibility, biomimetic qualities, biodegradability, and facile control over chemical functionalities. Such attributes position polymers as highly promising candidates for drug delivery systems. By employing precisely biodegradable polymer carriers, the duration of drug release can be finely tuned, spanning from several days to extended periods of weeks or even months. Polymer biodegradability plays a pivotal role in orchestrating the mechanism of drug release, involving an intricate interplay between diffusion processes, polymer erosion, and the controlled liberation of drugs. The formulation of polymer-based nanocarriers can be accomplished using a range of polymers, encompassing synthetic, semi-synthetic, and natural variants, contingent upon the specific demands of the application. Furthermore, these polymer-based nanocarriers hold considerable promise for the targeted delivery of biomolecules—encompassing proteins, drugs, genes, and growth factors—to various anatomical regions such as the cornea, regions of inflammation, and the central nervous system, even while contending with the blood–brain barrier [84]. Polymer selection and the methods employed in preparing polymer-based nanocarriers are contingent upon variables such as the nature of the drug, desired delivery profiles (controlled or rapid), and the intended target tissue. Natural polymer-based nanocarriers exhibit exceptional biocompatibility and biodegradability, rendering them suitable for applications involving soft tissues like nerves, cartilage, muscles, and the cornea. They are also applicable in wound dressing and drug delivery scenarios. Conversely, synthetic/semi-synthetic polymeric nanocarriers have found utility in achieving sustained and prolonged controlled drug release [85]. Achieving effective dosing, minimizing loss, ensuring minimal invasiveness, upholding patient compliance, and reducing systemic absorption stand as crucial considerations in the advancement of ocular drug delivery systems. 

#### 2.2.1. Gelatin Nanoparticles

**Figure 5:**
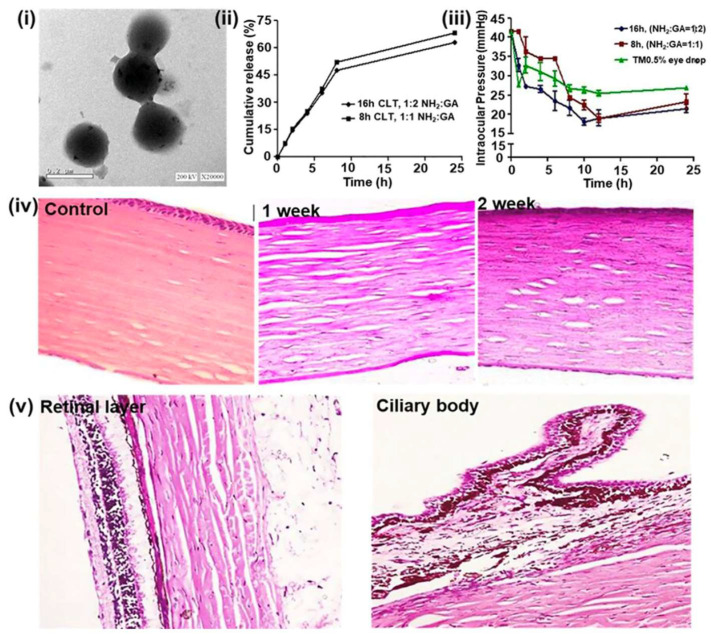
Natural polymer-based nanocarriers in drug delivery and safe in vivo efficacy. Transmission electron microscope image of timolol maleate-loaded gelatin nanoparticles: (**i**) Gelatin nanoparticles; (**ii**) timolol maleate release profile from the gelatin nanoparticles; (**iii**) IOP profile for 5.5 g of plunger drug-loaded gelatin nanoparticle formulation vs. 0.5% commercial product; (**iv**) H&E staining of corneal tissue of the control group after 1 and 2 weeks of treatment; and (**v**) H&E staining of the retinal layer and the ciliary body after treatment with drug-loaded gelatin nanoparticles at 14 days. Reproduced with permission from Elsevier from Ref. [86].

Gelatin is derived from the hydrolysis of collagen in either an acidic or alkaline medium. This substance has been extensively utilized as a biomaterial in various formats, such as hydrogels, membranes, microparticles, electrospun nanofibers, and nanoparticles, for applications in drug delivery and tissue engineering within the biomedical field. Shokry et al. fabricated gelatin nanoparticles loaded with timolol maleate using the double desolvation technique with glutaraldehyde (GA) crosslinking. This approach yielded optimized nanoparticles of approximately 205 nm in size and an entrapment efficiency of 74.72% (Figure 5i). Drug-loaded gelatin nanoparticles displayed a two-step drug release profile: a rapid initial release followed by a controlled release phase (Figure 5ii). Drug-loaded gelatin nanoparticles were compared to a commercially available timolol formulation in albino rabbits. This study concluded that nanocarriers exhibited superior efficiency in reducing IOP in a sustainable manner (Figure 5iii). Histological analysis using H&E staining indicated moderate edema of grade II after one week of treatment, while two weeks of treatment demonstrated mild edema of grade I (Figure 5iv). Additionally, the H&E results for the control, retinal layers, and the ciliary body exhibited a normal structure following two weeks of treatment with the drug-loaded particles (Figure 5v) [86]. In a separate study, Pérez et al. introduced a hybrid system of gelatin nanoparticles and hydroxypropyl methylcellulose (HPMC) for ocular topical administration of timolol maleate [87]. Notably, the gelatin nanoparticles exhibited a similar antihypertensive effect compared to a commercially available 0.5% timolol maleate formulation, despite utilizing a five-fold lower drug concentration in the in vivo study.

#### 2.2.2. Chitosan Nanoparticles

Chitosan, a naturally occurring polymer derived from sea creatures like crabs, shrimp, crustaceans, fungi, and insect exoskeletons, is primarily obtained through the deacetylation process of its parent polymer, chitin, which is abundant in nature. Chitin, in its original form, is insoluble in most organic solvents, whereas chitosan, having undergone deacetylation, is soluble in acidic aqueous environments with a pH of 6.0 or below, primarily due to the quaternization of its amine groups (with a pKa of 6.3) [88]. Chitosan, a cationic polymer, offers unique properties that can enhance drug delivery to the eye, including mucoadhesion [89], biocompatibility [90], and the ability to form nanoparticles that improve drug retention and bioavailability. Chitosan improves permeability by relaxing the tight connections between cells [91]. This versatile polymer can be employed to fabricate a range of structures, including microparticles, nanoparticles, film membranes, electrospun nanofibers, 3D scaffolds, and hydrogels, with applications spanning wound healing, drug delivery, antibacterial interventions, and tissue regeneration [92]. The chitosan nanoparticles are created through ionic or covalent crosslinking, emulsification, precipitation, or a mix of these methods [93,94]. A distinctive trait of chitosan is its positive surface charge, resulting from the abundance of amino groups, leading to the formation of various salts [94]. This positive charge enables chitosan to form strong electrostatic interactions, via charge-charge attractions, with negatively charged corneal mucin, facilitating prolonged bioavailability [95]. For instance, Kao et al. synthesized chitosan–carbopol nanoparticles loaded with the antiglaucoma drug pilocarpine and assessed the sustained release of the drug in both in vitro and in vivo contexts. The chitosan–carbopol nanoparticles exhibited a gradual release of pilocarpine, outperforming other formulations such as free pilocarpine, gel, and liposomes. In vivo miotic tests on rabbits demonstrated lasting pupil diameter reduction from the chitosan–carbopol nanoparticles [96]. Chitosan nanoparticles have found utility in encapsulating various drugs for glaucoma treatment. Katiyar et al. devised chitosan nanoparticles containing dorzolamide, which were dispersed in alginate gel to achieve prolonged, controlled release. Ex vivo permeation results indicated the superior performance of the in situ gel nanoparticles over available market solutions for glaucoma management [97]. Similarly, Papadimitriou et al. utilized chitosan nanoparticles to encapsulate pramipexole hydrochloride for oral and dorzolamide hydrochloride for ocular formulations. These formulations exhibited mucoadhesive properties that yielded sustained drug release [98]. Cho et al. introduced hexanoyl glycol chitosan as a platform for preocular drug delivery targeting glaucoma. This formulation demonstrated prolonged retention on the preocular surface, enhancing drug bioavailability [99]. In another study, Lin et al. developed chitosan-poly(acrylic acid) nanoparticles loaded with pilocarpine, showcasing controlled drug release in both in vitro and in vivo settings [100]. Chitosan nanoparticles can be clubbed with different drug delivery systems for stimuli-triggered drug delivery systems. Maulvi et al. aimed to design stimuli-sensitive (lysozyme-mediated) chitosan nanoparticles loaded into contact lenses to provide controlled ocular drug delivery. Chitosan nanoparticles were prepared using the ionotropic gelation method with sodium tripolyphosphate (TPP) as a crosslinking agent. To fabricate the contact lenses, a free radical polymerization technique was employed using a monomer solution comprising 2-hydroxyethyl methacrylate (HEMA), tris(hydroxymethyl)aminomethane (TRIS), N-vinyl-2-pyrrolidone (NVP), ethylene glycol dimethacrylate (EGDMA), and dimethylacrylamide (DMA), with a photoinitiator included to initiate and control the polymerization reaction. TM-Cht-NPs were spherical with some aggregation, averaging 190 nm in size and having a neutral zeta potential (+0.12 mV). Entrapment efficiency of timolol maleate was 53.36%. Conventional soaked lenses (SM-TM-CL) exhibited a high burst release of 78.25 µg (86.6% of total cumulative release) within 30 min, demonstrating a limitation of direct soaking. Direct timolol maleate laden lenses (DL-TM-CL) experienced significant drug loss during extraction (23.43 µg) and sterilization (21.85 µg), resulting in a total cumulative loss of 45.28 µg. In the presence of lysozyme (0.2 mM), TM-Cht-NPs lenses demonstrated a controlled release of 19.85 µg of timolol maleate over 120 h, with a release rate of 93–30 ng/h from 24 to 120 h. This confirms the enzymatic activity of lysozyme in breaking down the chitosan nanoparticles to release the drug. The eye drop group showed a reduction in IOP by 5.56 ± 0.21 mmHg, but the therapeutic effect declined after 6 h and returned to baseline within 12 h, resulting in an average reduction of 3.31 mmHg over 120 h with a "peak and valley" profile. In contrast, TM-NPs-CL contact lenses achieved a reduction in IOP by 5.66 mmHg with a prolonged effect lasting up to 120 h, maintaining an average reduction of 3.66 mmHg [101]. Rawat et al., developed nebivolol-loaded lecithin-chitosan hybrid nanoparticles (NEB-LCNPs) for improved ocular delivery in glaucoma treatment. The NEB-LCNPs were further incorporated into a dual-responsive in situ gel, which was characterized for various properties, including physicochemical characteristics, rheological behavior, stability, in vitro dissolution, and ocular in vivo studies to evaluate its effectiveness. The optimized nebivolol-loaded lecithin-chitosan hybrid nanoparticles (NEB-LCNPs) achieved a mean particle size of 170.5 ± 5.3 nm and a drug loading of 10.5 ± 1.2%, indicating a successful formulation for improved ocular delivery in glaucoma treatment. The NEB-LCNPs-ISG exhibited a two-fold increase in aqueous humor exposure, as evidenced by the pharmacokinetic studies, suggesting improved therapeutic efficacy for glaucoma management [102]. In another study, Wang et al., developed a multilayered sodium alginate-chitosan (SA-CS) hydrogel ball (HB), which encapsulates timolol maleate (TM) and levofloxacin in different layers to achieve sustained drug release for effective glaucoma treatment. The hydrogel ball utilizes zinc oxide-modified biochar (ZnO-BC) to enable photothermal conversion, allowing for on-demand regulation of drug release when stimulated by near-infrared irradiation. The multilayered sodium alginate-chitosan hydrogel ball (HB) demonstrated a sustained release of timolol maleate (TM) for over 2 weeks in vitro, effectively reducing intraocular pressure (IOP) in vivo [103]. In a different investigation, nanoparticles linked with hyaluronic acid, chitosan, and latanoprost (HA-CS-latanoprost link NP) were assessed for lowering intraocular pressure (IOP), showing greater efficacy than standard latanoprost and Xalatan. The maximum IOP reduction observed was 43% for the HA-CS-latanoprost link NP. The formulation of the HA-CS-latanoprost link NP is more efficient in decreasing IOP than the medication on its own [104]. However, despite these advantages, the use of chitosan and similar natural polymers presents several drawbacks. These include batch-to-batch variability due to differences in source material, limited mechanical strength, and instability under physiological pH, which compromise the reproducibility and robustness of formulations. Additionally, chitosan is only soluble under acidic conditions, limiting its application in neutral or alkaline environments such as the tear film. The potential for immunogenicity and slower degradation rates in certain modifications may also pose challenges for long-term ocular safety and regulatory approval.

#### 2.2.3. Hyaluronic Nanoparticles

Hyaluronic acid (HA) stands as another natural polymer, remarkable for its exceptional biocompatibility, biodegradability, susceptibility to chemical modifications, and minimal immunogenicity. These attributes have positioned it as a valuable biomaterial for diverse applications encompassing therapeutic and cell delivery, wound healing, and the regeneration of soft tissues. A study by Aragona et al. delved into the impact of topical sodium hyaluronate-containing artificial tears on 86 patients with moderate to severe dry eye, revealing that this formulation exhibited the potential to ameliorate the compromised ocular surface associated with dry eye syndrome [105]. Another endeavor by Fuente et al. involved the synthesis of hyaluronic acid-chitosan nanoparticles, unveiling a mechanism of effectiveness in gene delivery to the cornea and conjunctiva [106]. The developed nanoparticle formulation showcased a capacity for robust transfection while preserving cell viability. Intriguingly, these HA-chitosan nanoparticles were internalized via endocytosis, facilitated by the hyaluronic receptor CD44, ultimately facilitating gene targeting and transfection on the ocular surface. Apaolaza et al. embarked on a different avenue, crafting solid lipid nanoparticles integrating protamine and HA for gene delivery [107]. HA, a naturally occurring polysaccharide, can interact with DNA and cationic molecules like protamine. Protamine is a small, arginine-rich protein that binds tightly to DNA, neutralizing its negative phosphate backbone and inducing condensation into toroidal or globular structures. HA can bind to cell surface receptors like CD44 or RHAMM, which are often associated with nondegradative uptake mechanisms (e.g., caveolae-mediated endocytosis). This pathway helps bypass lysosomal degradation, enhancing the chances of DNA remaining functional. The study demonstrated effective transfection ability in both ARPE-19 retinal cells and HEK-293 kidney cells [108].

#### 2.2.4. PLGA- and PCL-Based Nanoparticles

The proliferation of synthetic polymer-based nanocarriers can be attributed to their cost-effective production, biocompatibility, manageable degradability, and extended drug release capabilities. Predominant among these synthetic polymer-based nanoparticles are PLA (poly(lactic acid)), PCL (polycaprolactone), and PLGA (poly(lactic-co-glycolic acid)). PCL is a versatile polymer used in various biomedical devices and implants. PCL is also used in synthesizing nanoparticles, microparticles, nanofibers, scaffolds, and injectable materials. A study by Lee et al. exemplified the fabrication of PCL-based polymer nanoparticles and nanocapsules loaded with the anti-glaucoma drug pilocarpine (PILO) utilizing the oil/water/oil double emulsion method [109]. These formulations exhibited similar sizes for both solid and hollow PCL nanocarriers, approximately 227 nm and 235 nm, respectively. While solid PCL nanocarriers displayed a burst release of the drug, the hollow PCL nanocarriers showcased sustained cumulative drug release over a span of 6 weeks, underlining the significance of their hollow structure. In glaucomatous eyes, both solid and hollow PCL nanocarriers manifested effectiveness after 5 days of treatment, but solely the hollow PCL nanocarriers maintained this efficacy up to 42 days. The therapeutic effect of drug release in the eye was further assessed through qualitative and quantitative evaluations of anterior chamber depth in glaucomatous rabbit eyes, revealing that the hollow PCL nanocarriers exhibited three times higher PILO loading capacity than solid PCL nanoparticles, consequently yielding a more potent therapeutic effect in glaucoma treatment. Another prominent synthetic polymer is PLGA, lauded for its biocompatibility, biodegradability, and FDA approval for therapeutic delivery and various biomedical applications. Comprised of diverse compositions of lactic acid (LA) and glycolic acid (GA), PLGA’s crystallinity and biodegradation vary based on its block structure and molar ratios. In a recent study by Pan et al., PLGA nanoparticles were synthesized using co-axial electrospray techniques and loaded with dexamethasone and melatonin for glaucoma treatment [110,111]. Encapsulation efficacy reached approximately 85%, and sustained drug release persisted for two weeks. Notably, R28 cell viability exceeded 90% at a concentration of 15 µg/mL for both dexamethasone and melatonin, indicating enhanced retinal penetration efficacy. This enhancement led to a significant reduction in intraocular pressure (IOP), thereby improving the management of glaucoma. Another compelling study by Nguyen et al. centered on hollow PLA nanoparticles with varying shell widths ranging from 10 to 100 nm, offering prolonged controlled release for glaucoma management [112]. Their findings indicated that nanoparticles with shell thicknesses between 70 and 100 nm had limited drug encapsulation capacity, while ultra-thin shell nanoparticles demonstrated accelerated drug release in comparison to thicker-shelled counterparts. The optimal shell thickness was determined to be 40 nm, facilitating long-term drug release spanning 56 days and proving effective in treating glaucomatous conditions in rabbit eyes. Sanchez-lopez et al., developed memantine-loaded nanoparticles (MEM-NP) using a double emulsion method, utilizing ethyl acetate as the organic solvent due to its partial water solubility and reduced toxicity compared to dichloromethane. This method involved studying the modifications of pH and composition of the two aqueous phases (w1 and w2) to optimize the formulation for ocular delivery. Design of experiments (DoE) was employed to achieve a suitable formulation, where it was found that smaller MEM-NP average sizes were obtained when the pH of the w1 phase was similar to the drug’s pKa (10.7). Additionally, encapsulation efficiency (EE) was maximized at w1 pH 11 and w2 pH 6.5, resulting in nanoparticles that incorporated 4 mg/mL of memantine, which were then used in subsequent experiments. The study presents a novel formulation of memantine-loaded PLGA-PEG nanoparticles that demonstrates an 80% encapsulation efficiency, with 0.35 mg/mL of memantine localized within the nanoparticle’s aqueous interior. This formulation was found to be better tolerated than free memantine in both epithelial and neuronal cell cultures in vitro. The topical administration of memantine-loaded nanoparticles was shown to be neuroprotective, significantly preserving retinal ganglion cell (RGC) density in a rodent model of ocular hypertension after twice-daily application, suggesting it as a safe, non-invasive, and effective strategy for glaucoma treatment [113].

#### 2.2.5. Dendrimers

Dendrimers belong to a class of polymers characterized by their highly structured, radially symmetrical branched configurations. They consist of three essential components: a central core, branched structures, and terminal functional groups. The inherent versatility of dendrimers allows for functionalization tailored to specific requirements, rendering them highly valued vehicles for efficient drug delivery [114]. Within the realm of diverse chemistries, poly(amidoamine) (PAMAM) dendrimers have found frequent use in ocular applications. A notable study by Lai et al. involved the formulation of pilocarpine-loaded gelatin grafted onto poly(N-isopropyl acrylamide) (PNIPAA) thermogel, demonstrating a remarkable encapsulation efficiency of approximately 62% [115]. Administered into the anterior chamber of rabbits with ocular hypertension (OHT) using a 30-gauge needle, this nanosystem resulted in increased drug concentration in the anterior chamber and sustained reduction in intraocular pressure (IOP) over a span of 2 weeks [114,116]. In vitro studies of drug release revealed an impressive cumulative release of around 95% of the payload within 14 days. Building on this foundation, the researchers further utilized PAMAM dendrimers as tethers to bind gelatin and PNIPAA, aiming to enhance biodegradation resistance, drug encapsulation efficiency, and release performance. In this extended investigation, both pilocarpine and ascorbic acid were concurrently encapsulated within the carrier. Following intracameral injection of a single dose of this drug delivery system (DDS) at 10% w/v concentration, the reduced IOP was sustained for over 80 days. Moreover, this DDS exhibited multifunctional attributes, including the mitigation of inflammatory mediators and the promotion of stromal collagen regeneration [117]. Yang et al., in order to achieve medicine adherence for glaucomatous patients, developed a hybrid dendrimer hydrogel/poly(lactic-co-glycolic acid) nanoparticle platform designed for the topical delivery of antiglaucoma drugs. This study developed two methods. Method 1 involves conjugating PEG diol to a PAMAM dendrimer, followed by the introduction of acrylate groups through reaction with acryloyl chloride. This allows for modulation of the loading density of PEG on the dendrimer surface and predetermined PEG chain lengths. Method 2 focuses on coupling one distal end of PEG diol to an acrylate group, which is then conjugated to the dendrimer. This method also utilizes UV light in the presence of a photoinitiator to create a cross-linked hydrogel network from the resulting photoreactive dendrimer macromonomers. The hybrid dendrimer hydrogel/poly(lactic-co-glycolic acid) nanoparticle platform (HDNP) demonstrated high drug absorption in ocular tissues and sustained control over intraocular pressure (IOP) for up to 4 days following a single topical administration of brimonidine and timolol maleate, significantly improving the potential for patient compliance in glaucoma management. The HDNP formulation achieved a 7.8-fold higher concentration of brimonidine in the aqueous humor and a 1.4-fold higher concentration in the cornea compared to traditional dendrimer hydrogel formulations, indicating enhanced bioavailability and prolonged IOP-lowering effects, which could reduce the frequency of dosing and improve long-term adherence to glaucoma treatment [118].

### 2.3. Vesicular Systems for Glaucoma Therapy

#### 2.3.1. Liposomes

Liposomes are spherical lipid vesicles that have gained significant attention as advanced drug delivery systems due to their unique composition, structure, and ability to overcome many challenges associated with conventional ocular drug delivery. Liposomes are primarily composed of natural or synthesized phospholipids and sphingolipids, along with cholesterol and hydrophilic polymers. They are characterized by their amphiphilic nature, meaning they can encapsulate both fat-soluble (lipophilic) medications within their double-layered membranes and water-soluble (hydrophilic) drugs in their inner aqueous core. Drug delivery liposomes typically have a single layer and range in size from 50 to 150 nanometers (nm). Liposomes interact with cells through various mechanisms, including surface adsorption, membrane fusion, phospholipid exchange, and endocytosis. Their phospholipid bilayer structure mimics natural cell membranes, which facilitates better interaction with ocular tissues and improves drug retention and release. The surface charge of liposomes significantly influences their effectiveness in ophthalmic applications. Positively charged liposomes, for example, demonstrate a higher likelihood of adhering to the negatively charged corneal surface compared to neutral or negatively charged liposomes. Cationic carriers are also anticipated to impede drug removal by tear flow due to increased solution thickness and interaction with negatively charged mucus. Studies have shown that positively charged unilamellar liposomes can significantly enhance the transport of penicillin G across isolated rabbit cornea, increasing trans-corneal flux by more than fourfold. Liposomes augment the delivery of anti-glaucoma medications like prostaglandin analogs and beta-blockers [46]. They enable effective combination therapies, such as timolol maleate and brimonidine tartrate, leading to a more pronounced reduction in intraocular pressure (IOP) compared to conventional treatments. Studies have shown that subconjunctivally injected liposomal latanoprost was more effective than daily eye drops in reducing IOP for approximately 50 days in rabbits. In another rabbit study, a single injection of latanoprost-loaded egg-phosphatidylcholine (EggPC) liposomes effectively lowered IOP for up to 90 days, achieving a greater reduction than daily topical administration. A first-in-man study involving six human subjects with ocular hypertension or primary open-angle glaucoma showed that a single subconjunctival injection of nanoliposomes (100 nm) containing latanoprost (POLAT-001) resulted in an immediate IOP reduction of 9 mmHg or more (≥20%). After three months, five out of six subjects maintained lower IOP compared to baseline. These patients tolerated the injection well, with only mild ocular discomfort reported immediately after. Natarajan et al. also demonstrated a sustained reduction in IOP in glaucomatous nonhuman primate models using latanoprost-loaded nanostructured lipid carriers (NLCs), with long-term efficacy for 120 days [119]. Zadeh et al., developed cationic liposomes as a targeted drug delivery system for the combination of timolol maleate and brimonidine tartrate in the treatment of glaucoma, highlighting the importance of multi-drug treatment systems in managing this chronic eye disease characterized by increased intraocular pressure and potential blindness. The study utilized the thin layer hydration method to create liposomal formulations containing timolol maleate and brimonidine tartrate, which are essential for improving drug delivery to ocular tissues in glaucoma treatment. Hydroxypropyl methylcellulose (HPMC) polymer was injected into the anterior chamber of rabbits to experimentally induce glaucoma. The study demonstrated that the liposomal formulation containing timolol maleate and brimonidine tartrate significantly reduced intraocular pressure in a glaucoma model compared to a simple aqueous solution formulation, with both formulations showing a significant reduction compared to the control group (*p* < 0.001). The optimized liposomal formulation exhibited a therapeutic effect in lowering intraocular pressure, maintaining its effectiveness for up to 90 h, indicating its potential as a suitable drug carrier for targeted and controlled drug delivery in glaucoma treatment [120]. Kouchak et al. formulated nanoliposomes encapsulating dorzolamide using the thin-film hydration technique, employing a phosphatidylcholine-to-cholesterol molar ratio of 7:4. The in vitro release profile showed a pronounced initial burst, with approximately 70% of the drug released within the first hour and nearly 95% released by six hours. This rapid release is likely due to the structural characteristics of the liposomal bilayers, which facilitate the swift diffusion of DRZ. In vivo studies conducted on rabbit eyes revealed that the nanoliposomal formulation provided enhanced and prolonged therapeutic outcomes compared to both plain DRZ solution and commercially available eye drops. The liposomes efficiently encapsulated DRZ in their aqueous core and adhered to the corneal surface, enabling controlled drug release. Furthermore, liposomal uptake via endocytosis may have contributed to drug transport across the corneal barrier, enabling release into the anterior segment of the eye [121]. Afify et al., designed self-assembling nanostructures composed of dorzolamide and L-α-Phosphatidylcholine to enhance the drug’s pharmacokinetic behavior and prolong its therapeutic efficacy in glaucoma management. These nanostructures were produced using a modified thin-film hydration approach, with optimization carried out through a response surface methodology. The formulations were evaluated for key characteristics, including drug loading efficiency, particle size, surface charge (zeta potential), and release profiles. The most effective formulation, prepared at a pH of 8.7 and incorporating different ratios of L-α-Phosphatidylcholine to dorzolamide, demonstrated improved drug concentrations in the aqueous humor and provided extended intraocular pressure reduction when compared to the commercial product Trusopt^®^. This research highlights the potential of such self-assembled delivery systems to enhance corneal penetration and offer sustained therapeutic benefits in glaucoma treatment [122]. Brugnera et al. prepared LAT-HA-LIP by combining latanoprost-loaded synthetic phosphatidylcholine liposomes with HA and osmoprotective agents (betaine and leucine) to enhance the hypotensive effect and protect the ocular surface. The liposomes were created using a mixture of specific phosphatidylcholine compounds, cholesterol, and α-tocopherol acetate. The LAT-HA-LIP formulation demonstrated a hypotensive effect that lasted 24 h longer than a marketed formulation after a single eye drop, indicating enhanced efficacy in reducing intraocular pressure (IOP) for glaucoma treatment. Additionally, the relative ocular bioavailability of LAT-HA-LIP was found to be almost three times higher than that of the marketed formulation, suggesting improved absorption and retention on the ocular surface [123]. Liposomes formulated using synthetic phosphatidylcholine and cholesterol were optimized for bimatoprost delivery. These liposomes had an encapsulation efficiency of 87.04% and a mean particle size of 306.78 nm. When encapsulated in a thermosensitive hydrogel, the system demonstrated zero-order drug release kinetics and reduced IOP for 18 h. This approach provided controlled drug delivery and improved patient compliance [124].

#### 2.3.2. Micelles

In recent times, the utilization of micelle-based nanocarriers has gained prominence for their potential in delivering lipophilic drugs. Nanomicelles, structured with hydrophobic cores and polar shells, arise from the assembly of amphiphilic polymeric surfactant block molecules under aqueous conditions [125]. These colloidal structures feature a soluble outer shell formed by the surfactants’ polar head, while the hydrophobic tails of the polymers remain within the core. This core provides a stable environment for encapsulating lipophilic drugs with high chemical stability [126]. During the design and production of nanomicellar formulations, various factors impact the final micelle characteristics, including size, surface charge, transport efficiency, drug release profile, and chemical stability under varying conditions like transport, storage, and specific physiological environments. These factors encompass the surfactant type, critical micellar concentration (CMC), additional additives, drug-to-surfactant ratio, administration routes, and storage conditions [127]. Gohar et al. utilized a two-level, two-factor full factorial design to optimize the formulation of self-assembled nanomicelles incorporating latanoprost (LAT), an anti-glaucoma drug. This design approach allowed for systematic evaluation of the formulation parameters affecting the nanomicelles. The optimized self-assembled latanoprost (LAT)-loaded nanomicelles demonstrated a spherical morphology with a size of 69 nm and an encapsulation efficiency of 77.5%, providing a sustained release of LAT over 12 h. In normotensive rabbits, the nanomicelles resulted in a significant reduction in intraocular pressure (IOP) by up to 40% for three days, which was notably longer than the IOP-lowering effect of XALATAN eye drops, which lasted only 24 h [128].

#### 2.3.3. Niosomes

A type of vesicular nanocarrier has also emerged as an effective platform for enhanced therapeutic drug delivery in clinical applications. Comprising a bilayer hydrophobic membrane encapsulating an aqueous core, niosomes can accommodate both hydrophobic and hydrophilic drugs. Momekova et al. recently provided a comprehensive overview of multifunctional niosome-based nanocarriers designed for advanced drug delivery applications [129,130,131]. The surfactant’s role is pivotal in stabilizing nanocarriers and facilitating drug loading and delivery. Zhang et al. introduced a novel approach by combining block polymer monomethoxy PEG (MPEG) with supramolecular cross-linkable polymers, such as alpha-cyclodextrin (α-CD), to create micellar hydrogels for ocular delivery of diclofenac [132]. Notably, micellar hydrogels with higher α-CD concentrations exhibited significantly slower release profiles, extending the release of diclofenac over 216 h compared to only 12 h from normal micelles. Critical micellar concentration (CMC), representing the concentration at which surfactants begin forming micelles, holds vital importance. Lower CMC values in polymers are preferable, as they ensure greater stability of nanomicelles upon dilution in the aqueous eye environment. Additional factors, including self-assembling surfactants, drug-to-surfactant ratios, administration routes, and storage conditions, also significantly influence nanocarrier development. The interlocking hydrogen bonds among the nanomicelle’s surfactants determine the system’s thermodynamic stability [133]. Moreover, compatibility between the drug and the surfactants forming the micelle is crucial in modulating drug loading and release profiles. Higher affinity leads to slower release rates, enabling sustained drug release [134]. Although the high lipophilicity of the corneal epithelium can hinder trans-corneal penetration of topically administered nanomicellar formulations, researchers are increasingly exploring the potential of conjunctival/scleral pathways to deliver these formulations to the posterior segment of the eye [135]. Concerning storage and transport, temperature variations may impact nanomicelles, potentially causing drug leakage. Freeze-drying nanomicellar formulations for dehydration allows storage for three months at 4 °C, resulting in an increase in CyA micelle size but maintaining formulation characteristics such as spherical shape, monodispersity, and absence of aggregation [136].

### 2.4. Miscellaneous Delivery Systems for Glaucoma Therapy

#### 2.4.1. Implants

**Figure 6:**
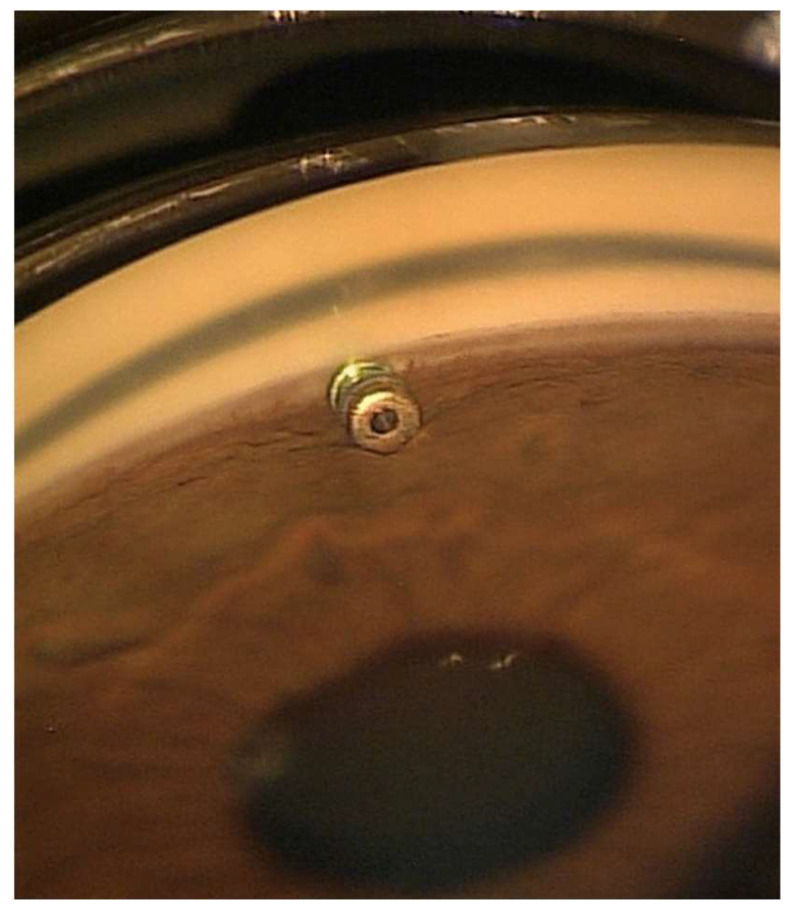
Travoprost intracameral implant anchored in the trabecular meshwork and sclera just anterior to the scleral spur and oriented parallel to the iris. Reprinted with permission from [145].

Intraocular implants can be made up of both biodegradable and non-biodegradable polymers and are primarily designed to offer controlled and localized drug release over extended periods. They help reduce the need for frequent intraocular injections, thereby mitigating associated side effects. Biodegradable implants are typically crafted from approved pharmaceutical polymers like polylactic acid (PLA), polycaprolactones (PCLs), polylactic-co-glycolic acid (PLGA), and polyglycolic acid (PGA). Non-biodegradable implants, on the other hand, are made from materials like silicone composite, polyvinyl alcohol (PVA), and ethylene vinyl acetate (EVA) [31,137,138]. The earliest attempts to achieve sustained drug release involved periocular devices placed beneath the eyelid. One notable example is Ocusert^®^, the first FDA-approved ocular implant available on the market [139]. The bimatoprost implant (Durysta^®^, developed by Allergan, Irvine, CA, USA) received FDA approval on March 5, 2020, for lowering intraocular pressure (IOP) in individuals diagnosed with open-angle glaucoma (OAG) or ocular hypertension (OHT). This implant represents the first FDA-approved sustained-release drug delivery system specifically for this indication. It consists of biodegradable polymers engineered to release bimatoprost at a consistent, steady rate over approximately 90 days. In preclinical studies involving dogs, a 15 µg dose of the implant resulted in the release of 80% of the total drug by day 51 and 99.8% by day 80, indicating efficient and predictable drug release kinetics [140]. When compared to conventional topical eye drops, the concentration of bimatoprost delivered via the implant was found to be approximately 4400 times higher at the iris–ciliary body [141]. Bimatoprost is a prostaglandin analog (PGA) known to lower IOP by enhancing aqueous humor outflow through both the trabecular meshwork (conventional pathway) and the uveoscleral pathway. Additional research using normotensive beagle dogs treated with a 30 µg implant indicated that bimatoprost may also help reduce episcleral venous pressure, potentially offering another mechanism for facilitating aqueous outflow [142]. The iDose TR implant (Glaukos Corporation, Aliso Viejo, CA, USA) is a titanium-based drug delivery system containing travoprost, designed for sustained intraocular release. The implant measures approximately 0.5 mm in width and 1.2 mm in length and is administered using a single-use inserter. It contains 75 micrograms of preservative-free travoprost, which is released over time through a nanoporous ethylene vinyl acetate (EVA) membrane. The release rate is governed by the thickness of the EVA membrane. There are two variants of the implant: a fast-eluting (FE) version and a slow-eluting (SE) version. Both versions are structurally identical except for the EVA membrane thickness. The SE version is approved for commercial use, whereas the FE variant is intended primarily for investigational studies. Implantation involves surgically positioning the device within the trabecular meshwork, where it is anchored to the sclera via a 0.6 mm long fixation element. In a phase II clinical study, Berdahl et al. evaluated the safety and efficacy of two different iDose TR implant designs in comparison with topical timolol 0.5% ophthalmic solution over a three-year period. The study demonstrated a notable reduction in mean baseline intraocular pressure (IOP) across all groups. Specifically, the slow-eluting (SE) implant achieved IOP reductions between 7.3 and 8.0 mmHg, while the fast-eluting (FE) version showed reductions ranging from 7.6 to 8.8 mmHg. For comparison, the timolol-treated group exhibited IOP reductions between 7.3 and 7.9 mmHg. Additionally, a greater proportion of patients receiving the implant (approximately 63% to 69% at the three-year mark) maintained adequate IOP control with the same or fewer adjunctive medications, compared to 45% in the timolol group. The implants were also associated with a favorable safety profile, supporting their potential as a long-term therapeutic alternative [143]. ENV515/Travoprost XR, developed by Envisia Therapeutics (Morrisville, NC, USA), is a biodegradable intraocular implant designed to provide a sustained release of travoprost for up to six months following anterior chamber injection (Figure 6). The implant is fabricated from poly(esteramide) (PEA) using PRINT® technology, which involves precision printing of the polymer, loading it with the active compound, and delivering it into the eye via a needle of appropriate gauge. Once implanted, the PEA matrix gradually degrades within the eye, following zero-order release kinetics, which enables a steady and controlled release of travoprost over time [144]. 

In preclinical testing on normotensive beagle dogs, administration of ENV515 led to an average 35 ± 3% reduction in intraocular pressure (IOP), reaching 6.4 ± 0.6 mmHg, maintained over a 24-week period. These studies also confirmed the implant’s stability and favorable safety profile. In a phase IIa clinical trial involving individuals with ocular hypertension, a single intracameral dose of ENV515 produced an average IOP reduction of approximately 6.7 ± 3.7 mmHg (equivalent to a 25% decrease from baseline) at 11 months post-implantation [145]. The treatment was generally well tolerated, although some participants experienced mild, dose-related transient hyperemia and eye redness [146]. PA5108, developed by PolyActiva (Melbourne, Australia), is a biodegradable, rod-shaped implant intended for intracameral administration using a 27-gauge needle. It is engineered to provide a sustained release of latanoprost over a six-month period. The implant initiates drug release immediately after placement, delivering a consistent daily dose without any initial burst effect, and follows a zero-order release profile. PolyActiva utilizes poly(ester), poly(triazole), and poly(urethane) systems—either individually or in combination—which degrade in a controlled manner to facilitate the steady release of the active pharmaceutical ingredient. In PA5108, latanoprost acid is covalently attached to a monomeric polymer subunit via a cleavable linker. The polytriazole hydrogel matrix supports continuous drug delivery for up to 20 weeks, after which the polymer undergoes biodegradation, completing the release process. Preclinical research involving ten dogs with glaucoma evaluated various latanoprost implant formulations, including PA5108. The implants led to significant reductions in intraocular pressure (IOP) over periods of 10, 19, and 34 weeks, outperforming both placebo devices and conventional latanoprost eye drops. The treatment was well-tolerated, with no signs of intraocular inflammation observed during the study. A phase I clinical trial is currently pending, which aims to further assess the safety, tolerability, and therapeutic potential of PA5108 in human subjects [147,148,149]. A summary of FDA-approved glaucoma implants is shown in Table 3.

#### 2.4.2. Contact Lens

**Figure 7:**
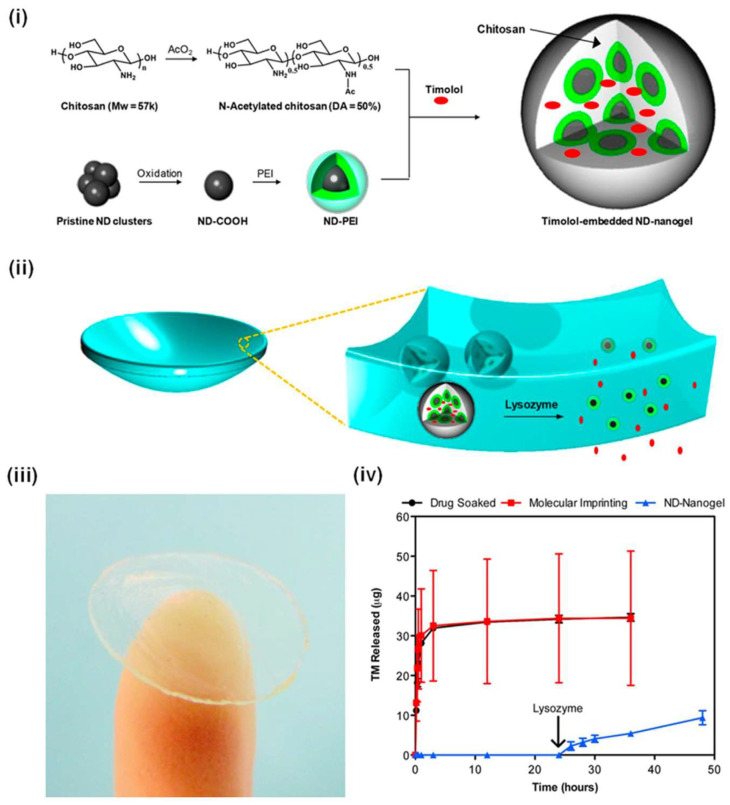
Lysozyme-activated drug-releasing contact lenses for therapeutic delivery: (**i**) Schematic representation of a lysozyme-activated active substance-releasing contact lens with ND nanogel and PEI, chitosan, and TM; (**ii**) action of the tear fluid lysozyme breaks the chitosan, decomposes the ND from nanogels, and TM release starts without affecting the lens structure; (**iii**) incorporation of ND nanogels in pHEMA to formulate contact lenses; and (**iv**) enzyme-activated TM release profiles from drug-soaked lenses, with molecular imprinting and ND embedded in nanogel-based lenses. Reproduced with permission from the American Chemical Society from Ref. [155].

Contact lenses (CLs) offer unique advantages for drug delivery due to their proximity to the cornea. One key advantage is that CLs extend the residence time of drugs to over 30 min, compared to just 5 min for eye drops. This prolonged contact significantly enhances drug bioavailability, potentially by as much as 50%. Notably, CLs are primarily composed of hydrogels, which have a high water content and are highly compatible with human tissues. Even with a water content of up to 99%, the oxygen permeability remains within a comfortable range, making extended wear feasible [150,151,152]. Because of these advantages, CLs are effective for treating ocular diseases like glaucoma, as they can release preloaded drugs over several days or even months. Various methods, including soaking, molecular imprinting, microemulsion, or nanoparticles, can be used to load drugs into CLs. Current manufacturing methods ensure that CLs have the appropriate thickness, water content, and optical properties. Importantly, the duration of CL application does not harm ocular tissues, and no adverse effects have been observed. Therapeutic CLs have the added benefit of reducing drug doses and associated side effects. They are especially beneficial for elderly patients who may struggle with frequent dosing regimens. These lenses can be made from materials such as poly(vinyl alcohol) (PVA), hydroxyethyl methacrylate (HEMA), N-vinyl-2-pyrrolidone (NVP), methacrylic acid (MAA), or poly(2-hydroxyethyl methacrylate) (pHEMA). Moreover, researchers have explored incorporating nanocarriers such as liposomes, micelles, and microemulsions into CLs [153,154]. Maulvi et al. developed a nanoparticle-loaded ring implant that could be placed between partially polymerized hydrogel contact lenses, enabling an extended release of timolol maleate (TM) for over 192 h without compromising the CL optical or physical properties [138]. Kim and colleagues have reported contact lenses embedded with nanodiamonds (NDs) that facilitate prolonged therapeutic effects through the controlled release of TM 299 triggered by enzymes. This was achieved by cross-linking polyethylenimine-functionalized NDs with chitosan, forming a nanogel with TM. This nanogel was then incorporated into a polyHEMA matrix to construct the drug-loaded NDs nanogel, which was finally integrated into a contact lens. Their resultant drug-eluting hydrogel-based contact lens exhibited enzyme-responsive (lysozyme) cumulative drug release, with approximately 9.14 μg released within a day (Figure 7) [155].

Currently, two types of drug-embedded contact lenses are being studied by Ciolino et al.,. One is a contact lens infused with latanoprost, while the other is a preservative-free contact lens containing bimatoprost (LL-BMT1, MediPrint Ophthalmics, San Diego, CA). Ciolino and colleagues [156] have explored the latanoprost-embedded contact lens. In a study involving glaucomatous eyes of cynomolgus monkeys, there was a statistically notable greater reduction in IOP with the contact lens releasing latanoprost compared to the latanoprost eye drops on days 3, 5, and 8. A phase II clinical trial assessed LL-BMT1 against topical timolol (0.5%, administered twice daily). They found a significant decrease in the average baseline IOP of 14% from the starting point at week one, escalating to 19% from the initial measurement by week 3, with no serious adverse effects reported [157]. In a separate study, Chauhan and co-researchers developed a temperature-sensitive hydrogel-based contact lens for targeted corneal drug delivery. Their approach involved using hydroxyl methyl methacrylate (HEMA) gels as a foundational material and embedding TM-loaded nanoparticles within the gel matrix to craft the contact lens. The in vitro release demonstrated controlled release of the drug over 2–4 weeks, a pattern that was subsequently validated in in vivo experiments [158]. Additionally, Lee et al. devised an ocular delivery system using contact lenses based on poly(2-hydroxyethyl methacrylate) (pHEMA) hydrogel. Their lenses incorporated vitamin E within the pHEMA-gel structure, leading to substantial improvements in the loading capacity for two hydrophobic glaucoma drugs (timolol and brimonidine) and a hydrophilic drug surrogate [159]. This enhancement translated to loading capacity increases of 19.1%, 18.7%, and 37.5% for the respective drugs. Salih et al. developed a novel technique for incorporating gold and silver nanoparticles into commercially available contact lenses using a process known as the “breathing-in/breathing-out” (BI-BO) method [160]. This innovative post-manufacturing approach maintains the original properties of the lens material, including its wettability and compatibility with biological tissues. The BI-BO process involves initially immersing hydrated contact lenses in an aprotic solvent to extract water. The dehydrated lenses are then rehydrated in a solution containing nanoparticles, enabling precise control over nanoparticle incorporation. This method is adjustable and has potential for large-scale application, as it also influences the optical characteristics of the lenses. In their study, Salih et al. showed that lenses embedded with AgNPs were capable of effectively blocking blue light within the 400–450 nm range—performance comparable to existing blue light-filtering eyewear. Additionally, lenses infused with AuNPs selectively filtered wavelengths around 522 nm, which could support red-green color vision correction, as also suggested in their earlier work [161,162].

#### 2.4.3. Hydrogels

Hydrogels are soft materials composed of water and polymers, which can be engineered through physical or chemical crosslinking [163,164]. The mechanical strength of hydrogels depends on molecular weight, concentration, and the type of crosslinking, be it physical, ionic, or chemical. Hydrogels offer controlled release due to the tunable physical and degradation properties of hydrogels, enabling them to protect encapsulated drugs or therapeutic molecules from degradation. The controlled drug release mechanism in hydrogels is regulated through network degradation, typically involving hydrolysis or enzymatic degradation of the polymer backbone [165,166,167]. A prodrug-hydrogel composite system was developed using timolol palmitate colloidal drug aggregates (CDAs) dispersed in a hyaluronan-oxime hydrogel. This system reduced IOP for at least 49 days in rat eyes, compared to 6 h for conventional timolol maleate. The hydrogel also minimized systemic side effects by reducing blood concentrations of timolol [168]. The injection of polyzwitterion hydrogel into the suprachoroidal space (SCS) expanded the uveovortex pathway, increasing aqueous humor drainage and reducing IOP for at least 6 weeks. This approach was well-tolerated and demonstrated minimal inflammatory reactions, making it a promising alternative to drug-based treatments [169]. A thermoresponsive hydrogel carrier combined with drug-loaded polymer microspheres was developed for long-term drug delivery. A single administration of this system reduced IOP in rabbits for 28 days, comparable to twice-daily brimonidine eye drops. The system was retained in the inferior fornix for the duration of the study, suggesting minimal systemic absorption [51]. HA-based nanocomposite hydrogels incorporating latanoprost-loaded liposomes were developed for sustained drug delivery. These hydrogels demonstrated prolonged drug release compared to liposomes or hydrogels alone, with in vitro release lasting over two weeks. The system also showed good biocompatibility and antibacterial properties, making it suitable for ocular applications [170]. These innovative hydrogel-based approaches hold promise for various ocular applications, including tissue regeneration and drug delivery, showcasing the versatility and potential of hydrogel materials in advancing ophthalmic treatments.

## 3. Future Prospective and Conclusions

Effective delivery of drugs is required to tackle the multifaceted nature of glaucoma, a chronic retinal neurodegenerative condition. Combining therapeutic agents targeting diverse pathophysiological mechanisms may prove more effective than monotherapy. Nanocarriers offer a solution for co-delivering multiple drugs with distinct physicochemical properties, a challenge unmet by conventional systems. For instance, Chan et al. devised a thermosensitive PLGA–PEG-PLGA copolymer capable of delivering hydrophobic and hydrophilic compounds (coumarin 6 and rhodamine B) simultaneously. This resulted in sustained drug concentrations for up to 4 weeks following a single subconjunctival injection. In the future, personalized multi-drug therapies tailored to each patient’s physiological profile using nano-/micro-drug carriers could become a standard treatment choice. The concept of a nano-in-micro (NIM) system involves integrating NPs into micro-matrices like hydrogels and microspheres. This hybrid approach harnesses the advantages of both nano- and micro-components while minimizing their respective drawbacks. The embedded NPs expand the total surface area for enhancing drug loading. For example, by combining NPs with limited biocompatibility with highly biocompatible polymers, the outer matrix safeguards both the NPs and drug within living tissues, enhancing drug release profiles and reducing toxicity.

Similarly, when dealing with mesoporous silica nanoparticles (MSN), which exhibit early cargo diffusion due to their open porous structure, embedding MSNs into a cyclosporine A-loaded PLGA-PEG-PLGA thermogel matrix can control the release. Smart stimuli-responsive delivery systems, categorized as "smart," can precisely release drug cargo in response to stimuli, whether exogenous (temperature, light, magnetic or electric fields, ultrasound) or endogenous (pH changes, enzyme activity). These systems enable site-specific delivery with minimal side effects, addressing a challenge for conventional NPs. Combining different stimuli-responsive components with NIM strategies can achieve programmed sequential release and multi-responsiveness. Although versatile smart stimuli-responsive DDSs have been developed for various diseases, their application in glaucoma remains underexplored.

While novel drug delivery systems offer significant advantages in enhancing ocular bioavailability and treatment adherence, their safety, feasibility of preparation, and cost-effectiveness are critical to their clinical success (Table 4). In particular, nanomaterials such as gold, silver, and iron oxide nanoparticles raise concerns regarding long-term accumulation in ocular tissues, oxidative stress, and potential immune responses, especially with chronic or repeated exposure. Although these materials enable targeted and sustained delivery, their biosafety profiles require thorough preclinical and long-term toxicity evaluations. 

From a practical standpoint, carriers like liposomes, micelles, and biodegradable polymers (e.g., PLGA, chitosan) are more feasible for scale-up and are supported by existing regulatory frameworks. In contrast, more complex systems, such as dendrimers or functionalized metallic nanoparticles, often involve labor-intensive synthesis methods and higher costs, which may limit their commercial viability unless they demonstrate clear clinical superiority. Additionally, implants and drug-eluting contact lenses, while more expensive initially, may prove cost-effective over time by reducing dosing frequency and improving adherence. These aspects must be considered alongside therapeutic efficacy when selecting appropriate platforms for glaucoma management. 

While this review provides a comprehensive overview of emerging drug delivery systems for glaucoma management, it is limited by the availability of long-term clinical data for many of the technologies discussed. Much of the current evidence is based on preclinical or early-phase studies, which may not fully capture the safety, scalability, or cost-effectiveness in real-world settings. Additionally, variations in formulation techniques, dosing strategies, and animal models make direct comparison across studies challenging. Future research should focus on standardized evaluation methods and long-term clinical trials to validate the translational potential of these novel systems.

In conclusion, glaucoma poses a global threat to vision across all age groups. Overcoming obstacles in glaucoma treatment, including patient non-adherence and limited drug bioavailability from topical eye drops, requires innovative solutions. Nanomaterial-based drug delivery holds significant promise by enabling sustained release, targeted delivery, improved bioavailability, reduced side effects, and enhanced efficacy. However, challenges such as scalable production, safety studies across diverse intraocular environments, and regulatory approval must be tackled for successful bench-to-bedside translation. Through multidisciplinary efforts, clinicians and patients can anticipate a broader range of therapeutic options in the years to come. 

## Figures and Tables

**Table 1:** Therapeutic treatment of glaucoma [6].

Class of Drug	Mechanism of Action	Examples of Drugs Belonging to Class	Dosage Form Available in Market	Side Effects	Approximate Monthly Cost of the Treatment
Prostaglandins analogues	Bind to the FP Receptor in the ciliary body, relaxes the uveoscleral muscle and thus increases fluid outflow	Bimatoprost, Latanoprost, Travoprost	Eyedrops	Irritation, redness of the eye, and discoloration of the iris, and surrounding skin [7]	USD 20–90 (generic) USD 120–180 (brand)
β-blockers	Blocks β-adrenoceptors in the ciliary body; this decrease in production of aqueous humor	Timolol, Levobunol Betaxolol	Eyedrops and Tablets	*Local effects:* irritation (burning, stinging, itching, tearing, redness), blurred vision, and dry eyes *Systemic effects:* fatigue, dizziness, slowed heart rate (bradycardia), low blood pressure (hypotension), breathing problems, and sexual dysfunction [8]	USD 10–60 (generic) up to USD 100 (brand)
Carbonic anhydrase	Inhibits carbonic anhydrase enzyme and ceases conversion of CO_2_ and H_2_O to HCO_3_, thus decreasing production of aqueous humor	Acetazolamide, Dorzolamide	Eyedrops and Tablets	*Local effects:* burning or stinging in the eyes, blurred vision, and eye irritation *Systemic effects:* fatigue, paresthesia, nausea [9]	USD 20–60 (drops) USD 15–30 (oral)
⍺-adrenergic agonist	Bind with ⍺-adrenergic receptors. Lowers the production of aqueous humor and increases trabecular and uveoscleral outflow	Clonidine, Brimonidine, Apraclonidine	Eyedrops and Tablets	*Local effects:* Redness, stinging, or burning sensation *Systemic effects:* Dry mouth, dry nose, mild chances of systemic hypotension [10]	USD 15–70 (generic) USD 120+ (brand)
Rho kinase inhibitors	Binds with the Rho kinase receptor present at the cornea, corneal endothelium, trabecular meshwork, and ciliary muscle. They increase trabecular meshwork outflow of aqueous humor, relaxing trabecular meshwork and Schlemm’s canal cells, potentially enhancing blood flow to the optic nerve	Netarsudil	Eyedrops	Conjunctival hyperemia, eye pruritus [11]	USD 200–250 (brand only)
Prostaglandin + β-blocker	Increases uveoscleral outflow + reduces aqueous humor production via β-blockade	Latanoprost + Timolol (Xalacom)	Eyedrops	Eye irritation, redness, potential cardiac issues [12]	USD 150–200
Carbonic anhydrase Inhibitor + β-blocker	Decreases aqueous production via carbonic anhydrase inhibition + β-blockade	Dorzolamide + Timolol (Cosopt)	Eyedrops	Bitter taste, burning, local inflammation, cardiac side effects [13]	USD 150–190
α-agonist + β-blocker	Reduces aqueous production and increases outflow (α-agonist) + β-blockade	Brimonidine + Timolol (Combigan)	Eyedrops	Dry mouth/nose, eye irritation, systemic hypotension [14]	USD 160–190
Rho kinase inhibitor + prostaglandin	Increases trabecular outflow (ROCK inhibition) + uveoscleral outflow via FP receptor	Netarsudil + Latanoprost (Rocklatan)	Eyedrops	Conjunctival hyperemia, eye discomfort [15]	USD 200–300
carbonic anhydrase + α-agonist	Dual reduction in aqueous production with a mild increase in outflow	Brinzolamide + Brimonidine (Simbrinza)	Eyedrops	Blurred vision, bitter taste, dry mouth [16]	USD 180–220

**Table 2:** Characteristics, advantages, and disadvantages of different drug delivery systems.

Drug Delivery System	Characteristics	Advantages	Disadvantages
**Inorganic Nanoparticles (e.g., AuNPs, AgNPs, Iron Oxide)**	Stable, high surface area, modifiable	Targeted delivery, sustained release, bioimaging potential	Potential cytotoxicity, long-term safety concerns
**Polymeric Nanoparticles (e.g., PLGA, PCL, Chitosan, Gelatin)**	Biodegradable, tunable release	Biocompatible, sustained delivery, tissue targeting	Manufacturing complexity, cost
**Liposomes**	Lipid bilayer vesicles	Encapsulate both hydrophilic and lipophilic drugs, biocompatible	Stability issues, burst release possible
**Micelles**	Amphiphilic nanostructures	Good for lipophilic drugs, sustained release	Dilution instability, short half-life
**Niosomes**	Non-ionic surfactant vesicles	Stable, low-cost alternative to liposomes	Lower encapsulation efficiency
**Contact Lenses**	Hydrogel-based, drug-loaded	Prolonged drug contact time, improved bioavailability	Handling issues, risk of contamination
**Implants (e.g., Durysta®, iDose TR)**	Biodegradable or permanent devices	Long-term release (weeks–months), improved compliance	Invasive, risk of complications, high cost

**Table 3:** Summary of FDA-approved glaucoma implants.

Brand Name of Implant	Mechanism/Delivery	Innovator Company	Price (USD, Approx.)	Unique Feature
iStent	Trabecular bypass	Glaukos (2012; iStent inject 2018)	~USD 1000–2000	Smallest implant; multi-placement option
Hydrus	Canal scaffold (90°)	Ivantis/Alcon (2018)	~USD 1000–2000	Dilates canal, tri-modal outflow
XEN Gel Stent	Subconjunctival bleb	Allergan (2016)	~USD 1000–2000	Gel-based; bleb-forming with smaller incision
iDose TR	Intracameral drug delivery	Glaukos (2023)	USD 13,950 per implant	Anchored implant; 24/7 drug release for years
Durysta	Biodegradable drug implant	Allergan (2020)	USD 1200–1500	Biodegradable; prostaglandin implant
Ahmed Valve	Valve-controlled shunt	New World Medical (2000s)	USD 1500–2500	Valve reduces hypotony risk
Baerveldt	Non-valved large-plate shunt	Johnson & Johnson	USD 1500–2500	Large surface area; long-lasting drainage
Ex-Press	Mini stainless shunt	Alcon	USD 800–1200	Predictable flow; small, under scleral flap

**Table 4:** Summary of metabolic fate, retention in ocular tissues, immunogenicity, and long-term safety of commonly used polymers.

System	Metabolism	Retention Time	Immune Response Risk	Potential Long-Term Effects
**Natural Polymers (Chitosan, Gelatin)**	Enzymatically degraded	Short (days to weeks)	Low	Safe, minimal toxicity
**PLGA/PCL**	Hydrolytic degradation to acids	Moderate (weeks/months)	Low–Moderate	Safe, but dependent on dose and formulation
**Inorganic Nanoparticles (Au, Ag, Fe)**	Not metabolized	Long (weeks to permanent)	Moderate–High	Possible accumulation, oxidative stress, inflammation
**Contact Lenses/Hydrogels**	Not metabolized (removed)	Hours to days	Low	Generally safe; hygiene-dependent
**Implants (Durysta®, iDose TR)**	Biodegradable or inert materials	Weeks to years	Low–Moderate	Risk of fibrosis, tissue remodeling, or device migration

## Data Availability

The data presented in this study is contained within this article.
